# Pneumococcal Meningitis during Pregnancy: A Case Report and Review of Literature

**DOI:** 10.1155/2007/63624

**Published:** 2007-04-02

**Authors:** Lisa M. Landrum, Angela Hawkins, Jean Ricci Goodman

**Affiliations:** Section of Maternal Fetal Medicine, Department of Obstetrics and Gynecology, Oklahoma University Health Sciences Center, Oklahoma City, OK 73190, USA

## Abstract

*Background*. Bacterial meningitis is a medical emergency for which prompt diagnosis and treatment are imperative to reducing the rate of death and long-term neurologic compromise. Few cases of meningitis have been reported during pregnancy, many of which had devastating outcomes for mother, neonate, or both. *Case*. A 38-year-old multigravida at 35 weeks of gestation presented with mental status changes, fever, and preterm contractions. Lumbar puncture revealed gram positive cocci consistent with *S. pneumoniae*. Patient was intubated and admitted to ICU where she was given antibiotics and adjunctive therapy with dexamethasone. Continuous fetal monitoring was utilized throughout her course of her hospitalization. Patient was discharged home after ten days in the hospital and had an uncomplicated vaginal birth after caesarean section (VBAC) at 38 weeks. Both she and the infant are doing well with no permanent neurologic sequelae. *Conclusion*. A review of literature indicates only isolated cases of pneumococcal meningitis being described during pregnancy. An extended period of time between onset of maternal illness and delivery appears to reduce the risk of neonatal transmission and improve both maternal and fetal outcomes.

## 1. BACKGROUND

Bacterial meningitis has an annual incidence of 4–6 cases per 100 000 adults and results in approximately 135 000 deaths worldwide each year. Since the development of the *Haemophilus influenzae* type B vaccine, the most common bacterial pathogen for community-acquired meningitis is *Streptococcus pneumoniae* which has a fatality rate from 19% to 37%. In patients that survive the initial insult, neurologic sequelae including seizures, hearing loss, impaired mental status, and/or cognition may occur in as many as 30% of all cases [1]. Local extension from contiguous extracerebral infection (e.g., otitis media, mastoiditis, or sinusitis) is a common cause. Patients with bacterial meningitis will usually present soon after the onset of symptoms with a classic triad of fever, neck stiffness, and altered mental status. Prompt recognition and treatment are key steps to reducing the morbidity and mortality associated with bacterial meningitis. In this case report, we describe a patient who presented during the third trimester of pregnancy with fever, mental status changes, and a subsequent diagnosis of bacterial meningitis. We will review the evaluation and management of this case as well as provide a literature review of other case reports of pneumococcal meningitis in pregnancy.

## 2. CASE

A 38-year-old multigravida with an intrauterine pregnancy at 35 weeks was found unconscious at her home and airlifted to the nearest tertiary care center. Throughout transport and upon arrival at the ED, her mental status alternated between combative and lethargic and was unable to provide any coherent information regarding her present condition or past medical history. Her family later reported that the patient had presented to her primary care physician five days earlier with complaints of left otalgia and low grade fever. Treatment was initiated with azithromycin for otitis media, but her pain continued to worsen precipitating an emergency room visit the day before her admission to the hospital. She received prescriptions for amoxicillin-clavulanate and opioids at this visit but had not yet started these medications. Her husband reported several bouts of emesis that same evening before her changes in mental status the following morning. Her primary obstetrician was also contacted and she verified an uncomplicated prenatal care with a term vaginal delivery in her first pregnancy, followed by a caesarian delivery for breech presentation in her second pregnancy. The patient had been counseled during this pregnancy regarding mode of delivery and opted for repeat caesarean section at 39 weeks.

On arrival in the ED, she was febrile to 38.7 C, with a pulse rate of 152 bpm, respiratory rate of 40, blood pressure of 147/80, and Glasgow coma score of 11 (4 for eye opening, 1 verbal, 6 movement). Fetal heart tones were noted to be 170 bpm with reassuring variability. Uterine contractions were noted every 2-3 minutes and initial sterile vaginal exam indicated cervical dilation of 4 cm with 50% effacement. The fetus was vertex in presentation with a size consistent for stated gestational age and a normal amniotic fluid index. The remainder of the physical examination was significant for purulent drainage from her left ear with marked erythema of both tympanic membranes. She was also noted to have decreased breath sounds at the base of the right lung. Complete blood count revealed a leukocytosis of 55 000 with 70% granulocytes and 22% bands. Chest X-ray indicated a possible infiltrate in the right middle and lower lobes. The patient was then intubated for airway protection, sedated, and given cetriaxone (2 g q 12 hours, IV) for empiric treatment of meningitis before being sent to the radiology suite. CT scan of the head revealed fluid collection in the middle ear and mastoid on the left. She was transferred to the intensive care unit and lumbar puncture obtained with 12 750 leukoctyes (80% PMN), glucose <5, and protein of 965. Preliminary blood and cerebrospinal fluid cultures indicated gram positive cocci in chains, consistent with *S. pneumoniae*. Vancomycin (1 g q 24 hours, IV) was then added for broader coverage, and dexamethasone (10 mg q 6 hours, IV) started as adjuvant therapy. Myringotomy at bedside revealed frank purulence in the middle ear which was evacuated and sent for culture. Continuous fetal and uterine monitoring was initiated with an experienced labor and delivery nurse posted at bedside for signs of fetal distress or continued preterm labor. This comprehensive level of care was continued until the level of sedation was decreased and the patient was extubated. Uterine contractions ceased in regularity after the first 24 hours with no further cervical change noted. Patient remained intubated until hospital day eight when her mental status had returned to baseline. At this time, she was transferred to the antepartum floor for two additional days of surveillance before discharge home. Final cultures from blood, CSF, and ear were consistent with *S. pneumoniae* with sensitivity to vancomycin. Patient completed the final four days of a 14-day course of vancomycin and ceftriaxone at home. Her obstetrical care was then returned to her primary obstetrician.

At 38 weeks gestation, the patient presented to her primary obstetrician with uterine contractions and was noted to be 9 cm dilated. She had a successful VBAC delivery of a male infant weighing eight pounds with APGAR scores of 8 and 9, at 1 and 5 minutes, respectively. Mother and child are doing well at the time of this writing, with no sequelae since delivery.

## 3. CONCLUSION

Bacterial meningitis is a medical emergency in which early diagnosis and treatment is imperative to prevent death and reduce long-term complications. Lumbar puncture is used to confirm the diagnosis in patients presenting with clinically suspected meningitis; however, imaging should be completed first in patients with new-onset seizures, an immunocompromised state, signs concerning for mass lesion or moderate-severe level of consciousness. If imaging is to be performed before lumbar puncture, empiric therapy should be initiated first as a delay in treatment can result in poor outcomes. The choice of initial antimicrobial therapy is based on the most common bacteria causing the disease according to the patient's age and the clinical setting and on patterns of antimicrobial susceptibility. With the worldwide increase in the prevalence of penicillin-resistant pneumococci, combination therapy with vancomycin plus a third-generation cephalosporin (ceftriaxone or cefotaxime) has become the standard approach to empirical antimicrobial therapy. Intravenous dexamethasone (10 mg q 6 hours for 4 days, IV) before or with the first dose of antibiotics has been shown to reduce the risk of death (14% versus 34%) and neurologic disability (26% versus 52%) in adults with pneumococcal meningitis when compared to placebo [2].

In a recent prospective study, the clinical features and prognostic factors were described in adults with bacterial meningitis. Risk factors for an unfavorable outcome were advanced age (>60 years), presence of otitis/sinusitis, absence of rash, low score on Glasgow coma score (<8), tachycardia (>120 bpm), a positive blood culture, an elevated erythrocyte sedimentation rate (>56), decreased platelet count (<180 000/mm^3^), low CSF white-cell count (<100/mm^3^), and causative species *S. pneumoniae* [3]. In the current case, the likely mechanism for transmission of the pathogen was through invasion of the central nervous system from a case of severe otitis media. Hence, our patient met a minimum of six of these ten criteria (tachycardia, otitis, absence of rash, positive blood culture, low CSF white-cell count, *S. pneumoniae*) placing her at increased risk for a poor outcome. In addition, it is not clear what impact the state of pregnancy has on prognosis for both mother and neonate.

A review of the literature was conducted using MEDine search and review of references cited. Key words utilized include pregnancy, meningitis, and pneumococcal. This revealed five single case reports and a small case series of women diagnosed with pneumococcal meningitis during pregnancy or the postpartum period. Lucas described 26 cases of pneumococcal meningitis in Nigerian during pregnancy (*n* = 15) or the immediate postpartum period (*n* = 11) during a time period from 1958 to 1962 [4]. The overall fatality rate for this group of women was 27% (7/26), and the rate of neurologic sequelae in survivors at time of discharge was 53% (10/19). The type of complications included hearing loss, severe emotional disturbance, aphasia, and hemiplegia. Of the 15 women diagnosed during pregnancy, 3 of the mothers died and the fetal loss rate from spontaneous abortion, stillbirth, and neonatal death was 47% (7/15). The high incidence of cases in Nigeria during this time period led the author to suggest that pregnancy predisposes women to pneumococcal meningitis. Although pregnancy does result in a diminished immune response, there is no data to conclude that there is increased risk specific to *S. pneumoniae*.

The single patient case reports are outlined in Table 1 (see [5–9]). The time interval between onset of maternal illness and the delivery of the neonate is less than 36 hours in three cases. Each of these cases resulted in either a fetal or maternal death, and in one instance both mother and infant died. In the other two cases, the time frame between onset of maternal illness and delivery was extended with a favorable outcome for both mother and infant. With the current case, discussion initially focused on whether it was prudent to move towards delivery, especially in light of her early signs of labor and her family's strong desire for a caesarian delivery. However, continuous fetal monitoring indicated a reassuring status throughout her hospitalization, and all signs of preterm labor had diminished leaving no indication for a premature delivery. It is difficult to draw conclusions from isolated reports, but the scant literature available suggests that an extended interval between the onset of maternal illness and delivery provides an important window of time for maternal and neonatal well-being.

## Figures and Tables

**Table 1:** Reported cases of pneumococcal meningitis during pregnancy.

Reference	Age	Gestational age at presentation	Parity	Presenting symptom	Maternal outcome	Fetal outcome

Hutchison et al. [6]	27	30 weeks	G2 P1	Premature rupture of membranes	Preterm vaginal delivery, followed by maternal death 36 hours later	Preterm female infant, 1750 g; *S. pneumoniae* sepsis; neonatal death 7 days later

Probst and Viviano [5]	21	6 weeks	G3 P2	Fever, neck pain, and headache	Hospitalized 17 days, followed by normal antepartum recovery and term delivery	Term female infant, 3040 g

Rennard [8]	23	27 weeks	G2 P1	Mental status changes	Hospitalized 16 days, followed by normal antepartum recovery and term delivery	Term female infant, 3180 g

Tempest [9]	35	40 weeks	G2 P1	Fever, uterine contractions and cough	Vaginal delivery 36 hours after admission, followed by 10-day hospitalization, normal recovery	Term male infant, 3100 g; *S. pneumoniae* meningitis; neonatal death 3 days later

Steiner et al. [7]	35	8 months	G5 P4	Mental status changes, fever, nuchal rigidity	Maternal death with postmortem cesarean delivery, 8 hours after admission	Preterm female infant; 2400 g; normal development at 5 years of age
